# Entanglement and entropy squeezing in the system of two qubits interacting with a two-mode field in the context of power low potentials

**DOI:** 10.1038/s41598-020-76059-5

**Published:** 2020-11-11

**Authors:** E. M. Khalil, K. Berrada, S. Abdel-Khalek, A. Al-Barakaty, J. Peřina

**Affiliations:** 1 Department of Mathematics and Statistics, College of Science, Taif University, P.O. Box 11099, Taif, 21944 Saudi Arabia; 2 Mathematics Department, Faculty of Science, Azhar University, Cairo, Egypt; 3 Department of Physics, College of Science, Imam Mohammad Ibn Saud Islamic University (IMSIU), Riyadh, Saudi Arabia; 4 The Abdus Salam International Centre for Theoretical Physics, Strada Costiera 11, Miramare, Trieste, Italy; 5 Mathematics Department, Faculty of Science, Sohag University, Sohag, 82524 Egypt; 6 Physics Department, The University College at Aljamoum, Umm Al-Qura University, Makkah, Saudi Arabia; 7 Joint Laboratory of Optics, Department of Optics, Palacký University, 17. Listopadu 50, 77207 Olomouc, Czech Republic

**Keywords:** Information theory and computation, Optical physics, Quantum physics, Physics

## Abstract

We study the dynamics of two non-stationary qubits, allowing for dipole-dipole and Ising-like interplays between them, coupled to quantized fields in the framework of two-mode pair coherent states of power-low potentials. We focus on three particular cases of the coherent states through the exponent parameter taken infinite square, triangular and harmonic potential wells. We examine the possible effects of such features on the evolution of some quantities of current interest, such as population inversion, entanglement among subsystems and squeezing entropy. We show how these quantities can be affected by the qubit-qubit interaction and exponent parameter during the time evolution for both cases of stationary and non-stationary qubits. The obtained results suggest insights about the capability of quantum systems composed of nonstationary qubits to maintain resources in comparison with stationary qubits.

## Introduction

Atom–photon interactions offer a practical way to manipulate and generate quantum entanglement, coherence and squeezing. The two-level atom inside a cavity field is the simplest case of the atom–photon interaction, described by the famous Jaynes–Cummings model (JCM)^1^. Since its introduction, the model has received great attention in the fields of quantum optics and laser physics for both experimental and theoretical studies^2–15^, and this interest is partly due to its apparent simplicity and, most importantly, to its remarkable predictions about the dynamical characteristics of subsystems. This model has come to be an inspiration for a wide range of generalizations inextricably linked to more general situations with realistic circumstances. Most of them concentrated mainly on multiple photon transformations and multiple fields^16,17^, noninteracting or interacting of a set of atoms in the same cavity^18,19^, described by the famous Tavis–Cummings model (TCM)^20^. In recent years, heightened interest has been paid to decoherence and quantum entanglement properties of light-matter interaction models ‘for bipartite and multipartite systems interacted with a cavity field and also with each other through dipole-dipole and Ising-like interactions^21–23^. In this regard, an important application focused on the resonant two-qubit JCM has been considered with the aim of excusing quantum protocols for clear Bell state differentiation of two qubits^24^.

One of the principal aspects of quantum physics is the quantum entanglement between two spatially separated objects sharing a common non-local wave function. Recently, entanglement, as a physical resource, is used to implement various tasks in information processing, communication and quantum computing^25–27^, including the information entropy^27,28^, the behavior of charge oscillations^29^, quantum cryptography^30^, etc. Several efforts have been carried out to quantify the entanglement between atoms and fields. Entanglement between photons and qubits has so far been exclusively studied at optical frequencies with single atoms^31^ and electron spins^32,33^, to interface stationary and flying qubits^34^, to implement quantum communication^35^ and to realize nodes for quantum repeaters^36^ and networks^37^. Advance rapid in the development of quantum superconducting circuit based on measuring quantum correlations between artificial atoms and itinerant photons has been considered^38–42^.

The concept of squeezed states has been widely examined for various radiation field schemes. Squeezing in a quantized electromagnetic field has received considerable attention and provided intriguing works in the literature^43^. This concept has expanded to atomic systems with analogous definitions of radiation fields^44–47^. The atom-photon interaction was used to determine the condition in which the squeezing effect would be present^48^. The aspect of atomic squeezing in a three-level atoms placed in a two-mode cavity is analyzed in the presence the dipole-dipole interaction^49^. The squeezed atomic model was considered on the basis of Raman scattering with a strong laser pulse to describe the transfer of the change in correlation between the atom and light^50^. The effect of the squeezing in the cases of nonlinear and optimal spin states was studied^51–53^. In addition, the experimental implementation for a set of V-type atoms was considered^54,55^. In all these cases, the atomic squeezing has been investigated in the context of the Heisenberg uncertainty relation (HUR). However, HUR cannot provide enough information on atomic squeezing, especially when the atomic inversion takes zero value^56^. This difficulty was overcome by applying the entropy uncertainty relationship (EUR)^57^.

This work is in keeping with the aforementioned spirit of putting forward another extension of the TCM, that is, an interactive version of it for the description of two identical nonstationary qubits. The qubits interact with each other via dipole-dipole and Ising-like interaction and with two-mode quantized field in the framework of pair coherent states of power-low potentials (PCSPLPs). The interaction characteristics of the proposed model is that the interaction between the qubit system and the field is considered to be a time-dependent function and the said field is associated with PLPs that provide energy differences. It is worth commenting that the set of results reported here, regarding the aforesaid nonlinear coupling scheme, may also be of some relevance in the light of novel experimental and theoretical research on optical simulation of the Tavis Cummings and Rabi models in current designs of architectures intended for quantum computation and communication. Motivated by these considerations, we strive to comprehend how the time-dependent coupling and exponent parameter influence the dynamics of qubits-fields entanglement, qubit-qubit entanglement and qubit squeezing in the presence of the dipole-dipole and Ising-like interaction.

The content of the manuscript is the following. In “Physical model”, the Hamiltonian system and general solution for a two-qubit system coupled to PCSPLPs with dipole-dipole and Ising interactions are introduced. “Measures and numerical results”, we present the numerical results of the possible effects of such features on the evolution of some quantities of current interest, such as population inversion, entanglement among subsystems and squeezing entropy. In “Conclusion”, some conclusions are given.

## Physical model

Let the Hamiltonian model of the system under study be described as follows:

1 $$\begin{aligned} H=H_F+H_{A}+H_{AF}+H_{AI}, \end{aligned}$$

where the constituent Hamiltonians are explicitly given by

2 $$\begin{aligned} H_F= & {} \sum _{L=A,B}^{{}} \hbar \omega _{L}{\hat{n}}_{L},\end{aligned}$$

3 $$\begin{aligned} H_{A}= & {} \sum _{L=A,B}^{{}}\frac{\hbar \Omega }{2} {\hat{\sigma }}_{z}^{(L)}, \end{aligned}$$

4 $$\begin{aligned} H_{AF}= & {} \sum _{L=A,B}^{{}}\hbar \lambda (t)\left( {\hat{a}}{\hat{b}}\ {\hat{\sigma }}_{+}^{(L)}+ {\hat{a}}^{\dagger }{\hat{b}}^{\dagger }\ {\hat{\sigma }}_{-}^{(L)}\right) , \end{aligned}$$

5 $$\begin{aligned} H_{AI}&=  {} \hbar \lambda _{D}\left( {\hat{\sigma }}_{+}^{(A)}{\hat{\sigma }}_{-}^{(B)}+ {\hat{\sigma }}_{-}^{(A)}{\hat{\sigma }}_{+}^{(B)}\right) +\hbar \lambda _{S}{\hat{\sigma }}_{Z}^{(A)}{\hat{\sigma }} _{Z}^{(B)}. \end{aligned}$$

here, $H_F$ and $H_A$ describe the energy operators of the two-mode field and qubits, respectively, the interplay between the qubit system and the quantized field is prescribed by $H_{AF}$, and $H_{AI}$ is the qubit-qubit interaction. The single field mode frequency is $\omega _L$, $\Omega _L$ is the qubit transition frequency, $\lambda (t)$ is the time-dependent coupling term, which is considered to be the same for both qubits, and $\lambda _D$ and $\lambda _S$ are the dipole-dipole and Ising parameters, respectively. The photon number operators ${\hat{n}}_A={\hat{a}}^{\dagger }{\hat{a}}$ and ${\hat{n}}_{B}=\hat{b }^{\dagger }{\hat{b}}$ where ${\hat{a}}^{\dagger }$ (${\hat{b}}^{\dagger }$) and $\hat{ a}$ (${\hat{b}}$) are, respectively, the photon creation and annihilation operators for the field mode *A* (*B*) such that $[{\hat{X}},{\hat{X}}^{\dagger }]={\hat{I}}$ ($X=a,b$), and, on the other side, ${\hat{\sigma }}_{+}^{(L)}({\hat{\sigma }}_{-}^{(L)})$ and ${\hat{\sigma }}_{z}^{(L)}$ $(L=A,B)$ indicate the standard qubit transition operators satisfying the commutation relations $[{\hat{\sigma }}_{z}^{(L)},{\hat{\sigma }}_{\pm }^{(L)}]=\pm 2\hat{ \sigma }_{\pm }^{(L)}$, $[{\hat{\sigma }}_{+}^{(L)},{\hat{\sigma }}_{-}^{(L)}]=\hat{ \sigma }_{z}^{(L)}$.

Large varieties of quantum systems can be described by PLPs^58–62^ through a convenient choice of the exponent parameter denoted by $\ell$. This parameter dictates and characterizes the level energy differences. For $\ell >2$, the level energy differences $\Delta E_n$ decrease with energy level *n*, but inversely so for $\ell <2$. For $\ell =2$, all $\Delta E_n$ are independent of *n*, the energy levels being equally spaced. Here, we introduce quantized fields for which the potentials and their corresponding energies are given by^63^

6 $$\begin{aligned} U(x,\ell )=U_0\left| {x\over a}\right| ^\ell ,\qquad E_n=\left( n+{\gamma \over 4}\right) ^{2\ell /(\ell +2)}, \end{aligned}$$

where $U_0(a)$ defines the dimension of energy (length).

Let the initial state is in a way such that the qubits are both in their corresponding excited state, $|++\rangle$, and the radiation field in two-mode PCSPLPs, $|z,\ell ,q \rangle$,

7 $$\begin{aligned} |\psi (0)\rangle =|++\rangle \otimes |z,\ell ,q \rangle , \end{aligned}$$

with the following correspondence^63,64^

8 $$\begin{aligned} |z,\ell ,q \rangle= & {} \left( \sum _{n=0}^{\infty }{\frac{|z|^{2n}}{\upsilon (n,\ell )\upsilon (n+q,\ell )}}\right) ^{-{\frac{1}{2}}}\sum _{n=0}^{\infty } \frac{z^{n}}{\sqrt{\upsilon (n,\ell )\upsilon (n+q,\ell )}}|n,n+q\rangle \nonumber \\= & {} \sum _{n=0}^{\infty }Q_{n}|n,n+q\rangle , \end{aligned}$$

where

9 $$\begin{aligned} \upsilon (n,\ell )=\prod _{k=1}^{n}\left\{ \left( k+{\frac{\varphi }{4}} \right) ^{{\frac{2\ell }{\ell +2}}}-\left( {\frac{\varphi }{4}}\right) ^{{ \frac{2\ell }{\ell +2}}}\right\} ,\quad \upsilon (0,\ell )=1. \end{aligned}$$

For the initial considerations, we work out that the wave function $|\psi (t)\rangle$ takes the form

10 $$\begin{aligned} |\psi (t)\rangle= & {} \sum _{n=0}^{\infty }(X_{1}(n,t)|e,e\rangle |n,n+q\rangle +X_{2}(n,t)|e,g\rangle |n+1,n+q+1\rangle \nonumber \\&+\, X_{3}(n,t)|g,e\rangle |n+1,n+q+1\rangle +X_{4}(n,t)|g,g\rangle |n+2,n+q+2\rangle ). \end{aligned}$$

So, it follows straightforwardly from the Schrödinger equation that the time-dependent coefficients can be determined and tackled this problem entails by numerical solution of the system of differential equations

11 $$\begin{aligned} i{dX\over dt}=\Lambda X, \end{aligned}$$

where

12 $$\begin{aligned} X=\left( \begin{array}{c} X_1 \\ X_2 \\ X_3 \\ X_4 \\ \end{array} \right) ,\quad \Lambda =\left( \begin{array}{cccc} 0 &{} \lambda (t)\nu _1(n) &{} \lambda (t)\nu _1(n) &{} 0 \\ \lambda (t)\nu _1(n) &{} \lambda _S &{} \lambda _D &{} \lambda (t)\nu _2(n) \\ \lambda (t)\nu _1(n) &{} -\lambda _D &{} -\lambda _S &{} \lambda (t)\nu _2(n) \\ 0 &{} \lambda (t)\nu _2(n) &{} \lambda (t)\nu _2(n) &{} 0 \\ \end{array} \right) , \end{aligned}$$

with

13 $$\begin{aligned} \nu _{j}(n)=\lambda \sqrt{(n+q+j)(n+j)}. \end{aligned}$$

The density matrix of the two-qubit system can be obtained by taking the trace over the radiation field

14 $$\begin{aligned} {\hat{\rho }}_{AB}(t)=Tr_{F}{\hat{\rho }}(t),\quad \text {with}\quad {\hat{\rho }}(t)=|\psi (t)\rangle \langle \psi (t)|, \end{aligned}$$

and for a single qubit system

15 $$\begin{aligned} {\hat{\rho }}_{A(B)}(t)&=  {} Tr_{B(A)}{\hat{\rho }}_{AB}(t). \end{aligned}$$

Based on the set of results, we are able to examine the influence of the PCSPLP, considering the case of an infinite square-well ($\ell \rightarrow \infty$, $\varphi =4$) potential, triangular well ($\ell =1$, $\varphi =3$), harmonic oscillator ($\ell =2$, $\varphi =2$), and time-dependent coupling on some properties of physical interest relating to the time evolution of qubit systems in the presence dipole–dipole and Ising-like interaction, such as the population inversion, qubits-field entanglement, qubit–qubit entanglement dynamics based on the negativity features and qubit squeezing with the help of the HUR.

## Measures and numerical results

### Population inversion

Now we are ready to consider the population inversion and discuss the behavior of the phenomena of collapses and revivals of the system Hamiltonian (1). It is known that the mathematical formula of population is the difference between the probability of finding the particle in excited and ground states. The population inversion *W*(*t*) of the qubits is given by

16 $$\begin{aligned} W(t)=\rho _{11}^{AB}+\rho _{22}^{AB}-\rho _{33}^{AB}-\rho _{44}^{AB}. \end{aligned}$$

**Figure 1:**
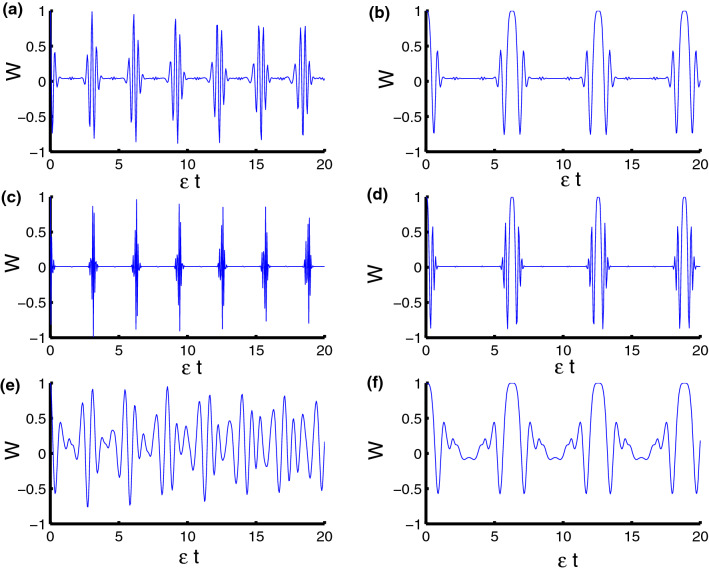
Dynamics of the population inversion as a function of time $\epsilon t$ with $z=8$ and $q=4$ for the case of $\lambda _D=\lambda _S=0$. **(a,c,e) ** correspond to stationary qubits $\lambda (t)=\varepsilon$, while **(b,d,f)** correspond to nonstationary qubits $\lambda (t)=\varepsilon \sin (t)$. Three PCSPLPs are considered: **(a,b)** correspond to harmonic potential, **(c,d)** correspond to triangular potential, and **(e,f)** for infinite well potential.

**Figure 2:**
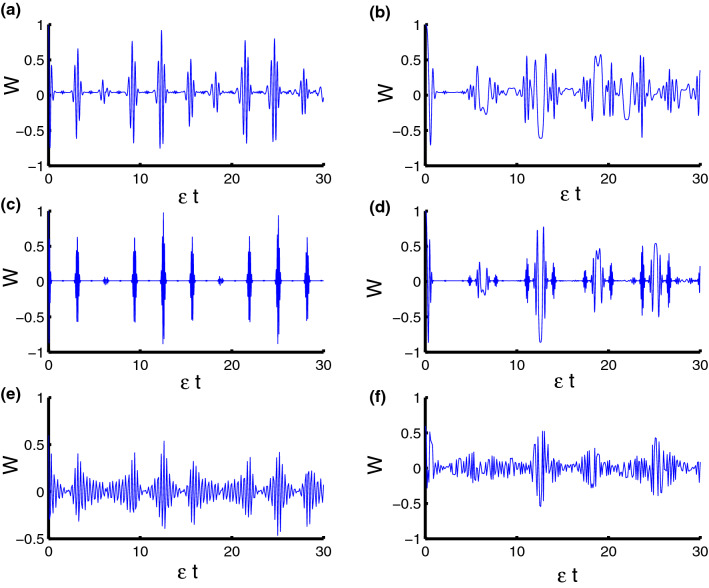
Dynamics of the population inversion as a function of time $\epsilon t$ with $z=8$ and $q=4$ for the case of $\lambda _{S}-\lambda _{D}=2.5-0.5=2$. **(a,c,e)** correspond to stationary qubits $\lambda (t)=\varepsilon$, while **(b,d,f)** correspond to nonstationary qubits $\lambda (t)=\varepsilon \sin (t)$. Three PCSPLPs are considered: **(a,b)** correspond to harmonic potential, **(c,d)** correspond to triangular potential, and **(e,f)** for infinite well potential.

In Fig. 1 the behavior of the function *W*(*t*) is drawn with fixed parameters $z=8$ and $q=4.$ For harmonic oscillator ($\ell =2$), neglecting the motion, we find that the function *W*(*t*) ranges between $-1$ and 1 around the horizontal axis. The collapse periods at $\frac{n\pi }{2}$ while the revivals at $n\pi$. We also note that there are oscillations having a small amplitude between periods of collapse, as observed by the Fig. 1a. After taking time dependence into account, we notice that the periods of collapse extend to double, whereas we find that the periods of revival decrease to twice as seen in Fig. 1b. For triangular well ($\ell =1$, $\varphi =3$), we exclude time dependence. We find that the revival periods are decreased and the fluctuation between the collapse periods in the previous case faded after taking into account triangular well. We also note that the amplitude of the oscillations expanded and became more regular compared to the previous case, see Fig. 1c. After adding dependence on time, we find once again that the periods of collapse increase while the periods of revival decrease and this result is consistent with the previous case, see Fig. 1d. For infinite square-well ($\ell \rightarrow \infty$, $\varphi =4$), we note that regular oscillations become chaotic and the amplitude of oscillations reduced after considering the case of infinite square-well. The phenomena of collapse and revival achieved in the previous case vanish in the case of infinite square-well, as shown in the Fig. 1e,f. In Fig. 2 we show the time evolution of the population inversion in the presence of the dipole-dipole and Ising interaction. From the figure, it is clear that the dynamical behavior of *W* is affected by the parameters $\lambda _D$ and $\lambda _S$ with respect to the physical parameters of the model. We observe that the qubit–qubit interactions lead to damage the periodicity of *W* accompanied with enhancement in the oscillations and a change in its time interval for which the revival and collapse phenomena occurring. Moreover, the presence of these interactions decreases the effect of the qubit–field coupling parameter $\lambda$.

### Qubits–field entanglement

To quantify the degree of the entanglement of the qubits–field state, we use the von Neumann entropy defined by

17 $$\begin{aligned} S_{AB}(t)=-\text {Tr}\left( \rho _{AB}\ln \rho _{AB}\right) . \end{aligned}$$

**Figure 3:**
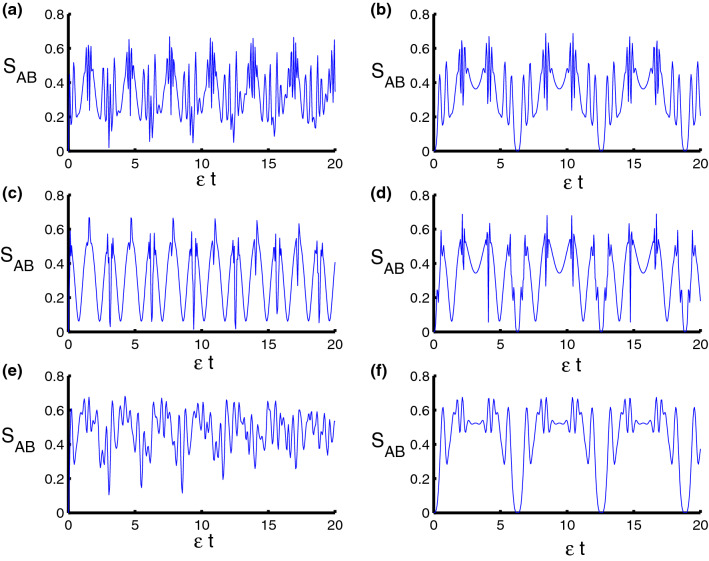
Dynamics of the von Neumann entropy as a function of time $\epsilon t$ with $z=8$ and $q=4$ for the case of $\lambda _D=\lambda _S=0$. **(a,c,e)** correspond to stationary qubits $\lambda (t)=\varepsilon$, while **(b,d,f)** correspond to nonstationary qubits $\lambda (t)=\varepsilon \sin (t)$. Three PCSPLPs are considered: **(a,b)** correspond to harmonic potential, **(c,d)** correspond to triangular potential, and **(e,f)** for infinite well potential.

**Figure 4:**
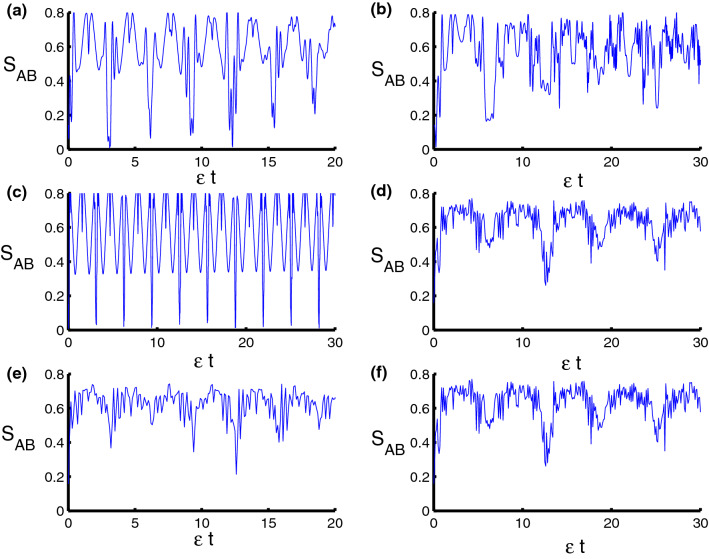
Dynamics of the von Neumann entropy as a function of time $\epsilon t$ with $z=8$ and $q=4$ for the case of $\lambda _{S}-\lambda _{D}=2.5-0.5=2$. **(a,c,e)** correspond to stationary qubits $\lambda (t)=\varepsilon$, while **(b,d,f)** correspond to nonstationary qubits $\lambda (t)=\varepsilon \sin (t)$. Three PCSPLPs are considered: **(a,b)** correspond to harmonic potential, **(c,d)** correspond to triangular potential, and **(e,f)** for infinite well potential.

In Fig. 3 the behavior of the von Neumann entropy is drawn with the same parameters as above. For harmonic oscillator ($$ \ell =2$$), after excluding time dependence, we see the entanglement fluctuating from weak to strong regularly, and the function $$S_{AB}(t)$$ reaches the smallest values when the extreme points of the population inversion. While the function $$S_{AB}(t)$$ reaches the maximum values from the center of the collapse areas as seen in the Fig. 3a. Fluctuations decrease and the function $$S_{AB}(t)$$ will reach the pure states ($$ S_{AB}(t)=0$$) regularly after taking time dependence into account as observed in Fig. 3b. For triangular well ($$\ell =1$$, $$\varphi =3$$) and in the absence of dependence on time, the speed of fluctuations decreased which means that entanglement becomes weak. It is pointed that function $$S_{AB}(t)$$ reaches the maximum and minimum values regularly compared to the previous case, see Fig. 3c. In general, the entanglement between parts of the system increases and the small values of the function ($$S_{AB}(t)=0$$) are reduced after taking time dependence into account as seen in Fig. 3d. For infinite square-well ($$\ell \rightarrow \infty $$, $$\varphi =4$$), the minimum values are raised up and then the function $$S_{AB}(t)$$ does not reach the pure state. We note that the fluctuations of the function $$ S_{AB}(t)$$ increased in the case of infinite square-well and the entanglement became strong compared to the previous two cases, see Fig. 3e. We note that the oscillations of the function $$S_{AB}(t)$$ become regular and reach the pure state periodically after adding the dependence on time in the interaction cavity as observed in Fig. 3f. In order to observe how the dipole-dipole and Ising interactions affect on the time variation of the qubits-field entanglement, clearly, in Fig. 4, we show the time evolution of function $$S_{AB}(t)$$ with respect to different values of the model parameters. We observe that the amount of the entanglement is strongly affected by the qubit–qubit interaction during the time evolution. The presence of the parameters $$\lambda _D$$ and $$\lambda _S$$ lead to enhance the oscillations of the function $$S_{AB}$$ and increase its value during the evolution. On the other hand, the existence of these parameters reduces the effect of the qubit-field coupling parameter $$\lambda $$ on the behavior of the entanglement.

### Qubit–qubit entanglement

In order to quantify the qubit-qubit entanglement, we use the negativity measure introduced as^65,66^:

18 $$\begin{aligned} N_{AB}=\frac{1}{2}\left\{ Tr\left[ \sqrt{\rho _{AB}^{T_{q}}(\rho _{AB}^{T_{q}})^{*}}\right] -1\right\} , \end{aligned}$$

where $\rho ^{T_{q}}$ is the partial transpose of $\varrho _{qf}$ for the qubit subsystem *q*, defined by

19 $$\begin{aligned} \left\langle k_{q},j_{f}|\varrho ^{T_{q}}|r_{q},l_{f}\right\rangle =\left\langle r_{q},j_{f}|\varrho ^{T_{q}}|k_{q},l_{f}\right\rangle . \end{aligned}$$

The negativity has a zero value for an entangled state and one value for maximally entangled states or EPR states.

**Figure 5:**
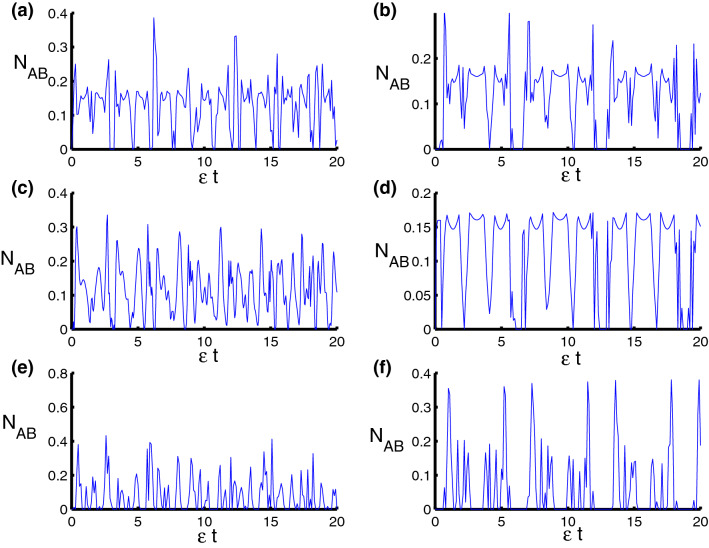
Dynamics of the negativity as a function of time $\epsilon t$ with $z=8$ and $q=4$ for the case of $\lambda _D=\lambda _S=0$. **(a,c,e)** correspond to stationary qubits $\lambda (t)=\varepsilon$, while **(b,d,f)** correspond to nonstationary qubits $\lambda (t)=\varepsilon \sin (t)$. Three PCSPLPs are considered: **(a,b)** correspond to harmonic potential, **(c,d)** correspond to triangular potential, and **(e,f)** for infinite well potential.

**Figure 6:**
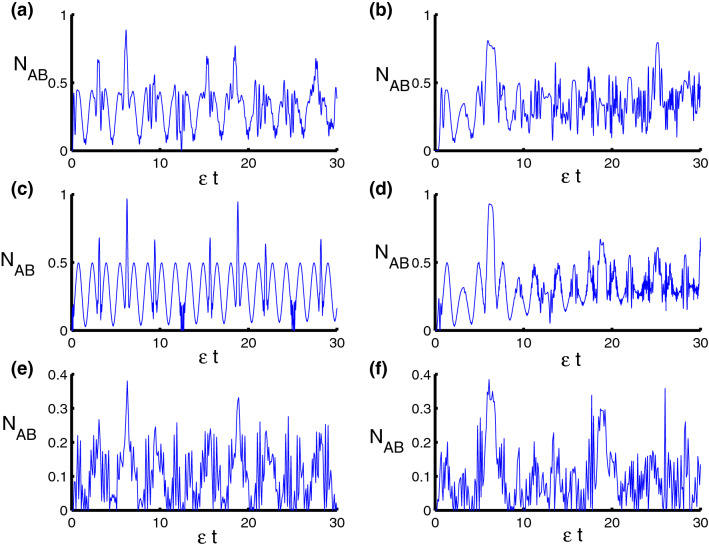
Dynamics of the negativity as a function of time $\epsilon t$ with $z=8$ and $q=4$ for the case of $\lambda _{S}-\lambda _{D}=2.5-0.5=2$. **(a,c,e)** correspond to stationary qubits $\lambda (t)=\varepsilon$, while **(b,d,f)** correspond to nonstationary qubits $\lambda (t)=\varepsilon \sin (t)$. Three PCSPLPs are considered: **(a,b)** correspond to harmonic potential, **(c,d)** correspond to triangular potential, and **(e,f)** for infinite well potential.

In Fig. 5, the negativity is potted to illustrate the time variation of the entanglement between the field and the two atoms by the above conditions. For harmonic oscillator ($\ell =2$), in general, the $N_{AB}(t)$ function fluctuates between the minimum (0) and the maximum (0.4), there is a partial entanglement between the field and the two atoms. We note that the function $N_{AB}(t)$ reaches the maximum values periodically at $n\pi$ while the function $N_{AB}(t)$ reaches the separation state at some points as shown in the Fig. 5a. After adding dependence on time, the previous chaotic oscillations become more uniform and the maximum values of $N_{AB}(t)$ decrease. This indicates that both the amount of entanglement and the points of separation state were reduced after taking time dependence into account as seen in Fig. 5b. For triangular well ($\ell =1$, $\varphi =3$) and in the absence of dependence on time, the function $N_{AB}(t)$ becomes more chaotic, the maximum values decrease and the entanglement becomes weak, as is evident from the Fig. 5c. The negativity decreases a lot after taking dependence on time and entanglement becomes weaker than the previous case, see Fig. 5d. For triangular well ($\ell =1$, $\varphi =3$) and in the absence of dependence on time, we note in this case the negativity is due to the differences between 0 and 0.4 and more regular than the previous cases. Minimum values are achieved for many periods, but the fluctuations have decreased compared to previous cases, see Fig. 5e. The fluctuations in negativity decrease and the periods of disentanglement between parts of the system increase after taking time dependence into account as observed in Fig. 5f. In order to observe how the dipole-dipole and Ising interaction affects the time variation of the qubit-qubit entanglement, clearly, the numerical results for the negativity in this case are displayed in Fig. 6. We show the negativity in terms of of $\epsilon t$ with respect to different values of the physical model. We observe that as we turn on the dipole-dipole and Ising interaction, the negativity is substantially increased at some specific times with an enhancement of the oscillations. This can be expected from the system’ Hamiltonian, whose interaction part involving the qubit operators naturally turns a separable state of the type $|++\rangle$ into an entangled state.

### Single qubit squeezing phenomena

The principle of uncertainty is one of the most fundamental assumptions in quantum theory, was first introduced by Heisenberg, which shows the limits of error in the common measurements of non-commutating operators in measuring quantum states^67–69^. In general the uncertainty principle for any two hermitian operators ${\hat{A}}$ and ${\hat{B}}$ obeys the relation $[{\hat{A}},{\hat{B}}]=i{\hat{C}},$ therefore the Heisenberg uncertainty inequality is given by,

20 $$\begin{aligned} \langle (\Delta {\hat{A}})^{2}\rangle \langle (\Delta {\hat{B}})^{2}\rangle \ge \frac{1}{4}|\langle {\hat{C}}\rangle |^{2}, \end{aligned}$$

where $\langle (\Delta {\hat{A}})^{2}\rangle =(\langle {\hat{A}}^{2}\rangle -\langle {\hat{A}}\rangle ^{2}).$ As one of important application is a Pauli operators ${\hat{\sigma }}_{X},$ ${\hat{\sigma }}_{Y}$ and ${\hat{\sigma }}_{Z}$ which are describes the interaction between a two-level atom and the electromagnetic field, such that $[{\hat{\sigma }}_{X},{\hat{\sigma }}_{Y}]=i{\hat{\sigma }}_{Z},$ therefore uncertainty can written as $\Delta {\hat{\sigma }}_{X}\Delta {\hat{\sigma }} _{Y}\ge \frac{1}{2}|\langle {\hat{\sigma }}_{Z}\rangle |$.

The single qubit entropy squeezing for the component ${\hat{\sigma }}_{\alpha }$^70^

21 $$\begin{aligned} E_{\alpha }(t)=\delta H({\hat{\sigma }}_{\alpha })-\frac{2}{\sqrt{\delta H(\hat{ \sigma }_{z})}}<0. \end{aligned}$$

where $\delta H({\hat{\sigma }}_{\alpha })=\exp \{H({\hat{\sigma }}_{\alpha })\}$, and $H({\hat{\sigma }}_{\alpha })$ is the Shannon information entropies of the atomic operators ${\hat{\sigma }}_{x}$, ${\hat{\sigma }}_{y}$ and ${\hat{\sigma }}_{z}$.

**Figure 7:**
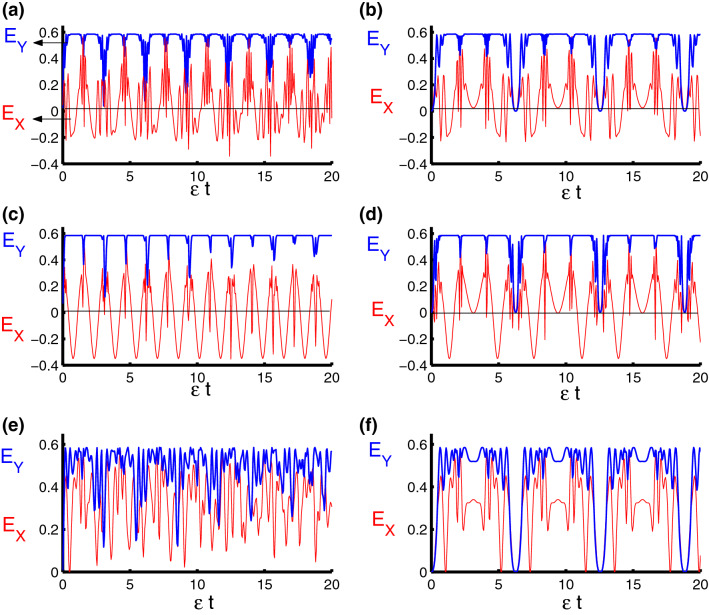
Dynamics of the entropy squeezing components, $E_X$ and $E_Y$, as a function of time $\epsilon t$ with $z=8$ and $q=4$ for the case of $\lambda _D=\lambda _S=0$. **(a,c,e)** correspond to stationary qubits $\lambda (t)=\varepsilon$, while **(b,d,f)** correspond to nonstationary qubits $\lambda (t)=\varepsilon \sin (t)$. Three PCSPLPs are considered: **(a,b)** correspond to harmonic potential, **(c,d)** correspond to triangular potential, and **(e,f)** for infinite well potential entropy squeezing components.

**Figure 8:**
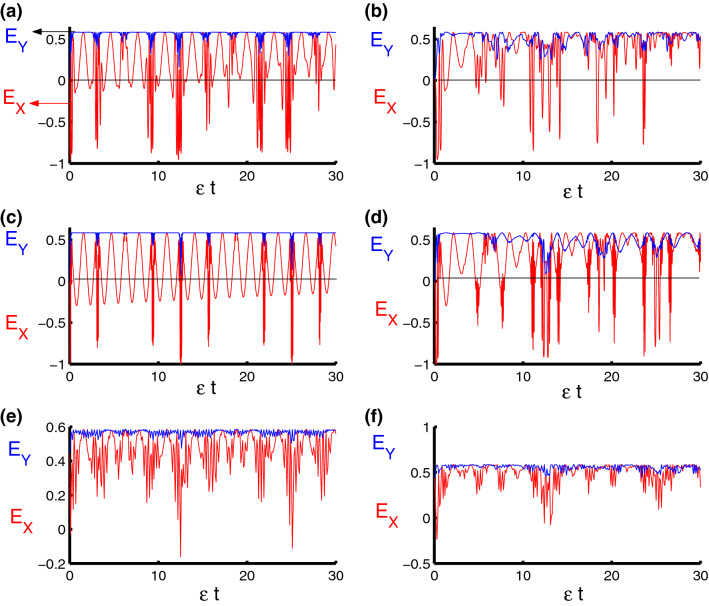
Dynamics of the entropy squeezing components, $E_X$ and $E_Y$, as a function of time $\epsilon t$ with $z=8$ and $q=4$ for the case of $\lambda _{S}-\lambda _{D}=2.5-0.5=2$. **(a,c,e)** correspond to stationary qubits $\lambda (t)=\varepsilon$, while **(d,f)** correspond to nonstationary qubits $\lambda (t)=\varepsilon \sin (t)$. Three PCSPLPs are considered: **(a,b)** correspond to harmonic potential, (c,d) correspond to triangular potential, and **(e,f)** for infinite well potential.

In Fig. 7, we display the entropy squeezing as a function of time considering the conditions as in the previous sections. Generally the squeezing is achieved with respect to $$ E_{X}(t)$$ and never with respect to $$E_{Y}(t)$$, in the first case we note that squeezing is achieved regularly and periodically before and after the center of the collapses regions as shown in the Fig. 7a. Squeezing areas decrease after adding time dependence to the interaction cavity. The squeezing occurs at the beginning and the end of the collapses periods and disappears in the middle of these periods with a comparison between Fig. 1b and 7b. In the second case, the squeezing periods increase and the maximum values increase to reach $$-0.4$$ periodically at $$\frac{n\pi }{4}$$ as seen in Fig. 7c. Once again, the squeezing decreases after adding dependence on time. The squeezing occurs at the beginning and the end of the collapse periods and disappears in the middle of these periods, see Fig. 7d. In the last case, the squeezing disappears, with and without depending on time, as seen in the Fig. 7e,f. In order to examine the dynamical behavior of the entropy squeezing of the qubit system in the presence of the qubit–qubit interaction, the time evolution of the entropies $$E_X$$ and $$E_Y$$ versus the dimensionless quantity $$\epsilon t$$ is displayed in Fig. 8 with respect to different values of the physical parameters of the model. The presence of the dipole–dipole and Ising interaction leads to reduce the squeezing effect and enhance the oscillations of the functions $$E_X$$ and $$E_Y$$ during the time evolution. On the other hand, the existence of these parameters decrease the effect of the qubit–field coupling parameter $$\lambda $$ on the behavior of the entropies.

## Conclusion

In summary, we have introduced a useful model describing the dynamics of two nonstationary qubits, allowing for dipole–dipole and Ising-like interplays between them, coupled to quantized fields in the framework of two-mode pair coherent states of power-low potentials. We have considered three particular cases of the coherent states through the exponent parameter taken infinite square, triangular and harmonic potential wells. We have examined the possible effects of such features on the evolution of some quantities of current interest, such as population inversion, entanglement among subsystems and squeezing entropy. We have shown how these quantities can be affected by the qubit–qubit interaction and exponent parameter during the time evolution for both cases of stationary and nonstationary qubits. Moreover, we have explored the dependence among the quantities on the main parameters of the physical model. The obtained results suggest insights about the capability of quantum systems composed of nonstationary qubits to maintain resources in comparison with stationary qubits.

## References

[1] Jaynes ET, Cummings FW (1963). Comparison of quantum and semiclassical radiation theories with application to the beam maser. Proc. IEEE.

[2] Wang Y, Wu JL, Song J, Zhang ZJ, Jiang YY, Xia Y (2020). Enhancing atom-field interaction in the reduced multiphoton Tavis-Cummings model. Phys. Rev. A.

[3] Fiscelli G, Rizzuto L, Passante R (2020). Dispersion interaction between two hydrogen atoms in a static electric field. Phys. Rev. Lett..

[4] Hood JD, Yu Y, Lin Y-W, Zhang JT, Wang K, Liu LR, Gao B, Ni K-K (2020). Multichannel interactions of two atoms in an optical tweezer. Phys. Rev. Res..

[5] Cortiñas RG, Favier M, Ravon B, Méhaignerie P, Machu Y, Raimond JM, Sayrin C, Brune M (2020). Laser trapping of circular Rydberg atoms. Phys. Rev. Lett..

[6] Chávez-Carlos J, López-del-Carpio B, Bastarrachea-Magnani MA, Stránský P (2019). Quantum and classical Lyapunov exponents in atom-field interaction systems. Phys. Rev. Lett..

[7] Scully Marlan O, Suhail Zubairy M (1997). Quantum Optics.

[8] Eberly JH, Narozhny NB, Sanchez-Mondragon J (1980). Periodic spontaneous collapse and revival in a simple quantum model. J. Phys. Rev. Lett..

[9] Cummings FW (1965). Stimulated emission of radiation in a single mode. Phys. Rev. A.

[10] Han Y, Xiao J, Liu Y, Zhang C, Wang H, Xiao M, Peng K (2008). Interacting dark states with enhanced nonlinearity in an ideal four-level tripod atomic system. Phys. Rev. A.

[11] Baghshahi HR, Tavassoly MK (2014). Entanglement, quantum statistics and squeezing of two $$\Xi $$-type three-level atoms interacting nonlinearly with a single-mode field. Phys. Scr..

[12] Cordero S, Recamier J (2011). Selective transition and complete revivals of a single two-level atom in the Jaynes-Cummings Hamiltonian with an additional Kerr medium. J. Phys. B.

[13] Cordero S, Recamier J (2012). Algebraic treatment of the time-dependent Jaynes-Cummings Hamiltonian including nonlinear terms. J. Phys. A.

[14] Chaichian M, Ellinas D, Kulish P (1990). Quantum algebra as the dynamical symmetry of the deformed Jaynes-Cummings model. Phys. Rev. Lett..

[15] Santos-Sanchez DL, Recamier O (2012). The f-deformed Jaynes-Cummings model and its nonlinear coherent states. J. Phys. B.

[16] Parkins AS (1990). Resonance fluorescence of a two-level atom in a two-mode squeezed vacuum. Phys. Rev. A.

[17] Joshi A, Puri RR (1990). Characteristics of Rabi oscillations in the two-mode squeezed state of the field. Phys. Rev. A.

[18] Joshi A, Puri RR, Lawande SV (1991). Effect of dipole interaction and phase-interrupting collisions on the collapse-and-revival phenomenon in the Jaynes-Cummings model. Phys. Rev. A.

[19] Chilingaryan SA, Rodrguez-Lara BM (2013). Searching for structure beyond parity in the two-qubit Dicke model. J. Phys. A.

[20] Tavis M, Cummings FW (1969). Approximate solutions for an N-molecule-radiation-field Hamiltonian. Phys. Rev..

[21] Hartmann MJ, Brand GSL, Plenio MB (2007). Effective spin systems in coupled microcavities. Phys. Rev. Lett..

[22] Torres JM, Sadurni E, Seligman TH (2010). Two interacting atoms in a cavity: Exact solutions, entanglement and decoherence. J. Phys. A.

[23] Porras D, Cirac JI (2004). Effective quantum spin systems with trapped ions. Phys. Rev. Lett..

[24] Torres JM, Bernad JZ, Alber G (2016). Unambiguous atomic Bell measurement assisted by multiphoton states. Appl. Phys. B.

[25] Wang X, Wilde MM (2020). Cost of quantum entanglement simplified. Phys. Rev. Lett..

[26] Klco N, Savage MJ (2020). Minimally entangled state preparation of localized wave functions on quantum computers. Phys. Rev. A.

[27] Nielsen MA, Chuang IL (2000). Quantum Computation and Quantum Information, Cambridge Series on Information and the Natural Sciences.

[28] Alber G, Beth T, Horodecki M, Horodecki P, Horodecki R, Rtteler M, Weinfurter H, Zeilinger A (2001). Quantum Information.

[29] Benatti F, Floreanini R, Realpe-Gomez J (2008). Entropy behaviour under completely positive maps. J. Phys. A.

[30] Horodecki, R., Kilin, S. Y. & Kowalik, J. *Quantum Cryptography and Computing: Theory and Implementation* (Nato Science for Peace and Sec, 2010).

[31] Blinov BB, Moehring DLL, Duan M, Monroe C (2004). Observation of entanglement between a single trapped atom and a single photon. Nature.

[32] Togan E (2010). Quantum entanglement between an optical photon and a solid-state spin qubit. Nature.

[33] Castelano LK, Fanchini FF, Berrada K (2016). Open quantum system description of singlet-triplet qubits in quantum dots. Phys. Rev. B.

[34] Wilk T, Webster SC, Kuhn A, Rempe G (2007). Single-atom single-photon quantum interface. Science.

[35] Olmschenk S, Matsukevich DN, Maunz P, Hayes D, Duan L-M, Monroe C (2009). Quantum teleportation between distant matter qubits. Science.

[36] Yuan Z-S, Chen Y-A, Zhao B, Chen S, Schmiedmayer J, Pan J-W (2008). Experimental demonstration of a BDCZ quantum repeater node. Nature.

[37] Ritter S, Nölleke C, Hahn C, Reiserer A, Neuzner A, Uphoff M, Mücke M, Figueroa E, Bochmann J, Rempe G (2012). An elementary quantum network of single atoms in optical cavities. Nature.

[38] Houck A, Schuster DI, Gambetta JM, Schreier JA, Johnson BR, Chow JM, Frunzio L, Majer J, Devoret MH, Girvin SM, Schoelkopf RJ (2007). Generating single microwave photons in a circuit. Nature.

[39] Mooney GJ, Hill CD, Hollenberg LCL (2019). Entanglement in a 20-qubit superconducting quantum computer. Sci. Rep..

[40] Tsujimoto M, Fujita S, Kuwano G, Maeda K, Elarabi A, Hawecker J, Tignon J, Mangeney J, Dhillon SS, Kakeya I (2020). Mutually synchronized macroscopic Josephson oscillations demonstrated by polarization analysis of superconducting terahertz emitters. Phys. Rev. Appl..

[41] Hofheinz M, Wang H, Ansmann M, Bialczak RC, Lucero E, Neeley M, O’Connell AD, Sank D, Wenner J, Martinis JM, Cleland AN (2009). Synthesizing arbitrary quantum states in a superconducting resonator. Nature.

[42] Eichler C, Lang C, Fink JM, Govenius J, Filipp S, Wallraff A (2012). Observation of entanglement between itinerant microwave photons and a superconducting qubit. Phys. Rev. Lett..

[43] Drummond PD, Ficek Z (2004). Quantum Squeezing.

[44] Wodkiewicz K (1981). Reduced quantum fluctuations in the Josephson junction. Phys. Rev. B.

[45] Agarwal GS, Puri RR (1990). Cooperative behavior of atoms irradiated by broadband squeezed light. Phys. Rev. A.

[46] Ashraf MM, Razmi MSK (1992). Atomic-dipole squeezing and emission spectra of the nondegenerate two-photon Jaynes-Cummings model. Phys. Rev. A.

[47] Kitagawa M, Ueda M (1993). Squeezed spin states. Phys. Rev. A.

[48] Civitarese O, Reboiro M (2006). Atomic squeezing in three level atoms. Phys. Lett. A.

[49] Civitarese O, Reboiro M, Rebón L, Tielas D (2010). Atomic squeezing in three-level atoms with effective dipole-dipole atomic interaction. Phys. Lett. A.

[50] Poulsen UV, Mølmer K (2001). Squeezed light from spin-squeezed atoms. Phys. Rev. Lett..

[51] Wang X (2001). Spin squeezing in nonlinear spin-coherent states. J. Opt. B: Quantum Semiclass. Opt..

[52] Rojo AG (2003). Optimally squeezed spin states. Phys. Rev. A.

[53] Wang X, Sanders BC (2003). Relations between bosonic quadrature squeezing and atomic spin squeezing. Phys. Rev. A.

[54] Dicke RH (1954). Coherence in spontaneous radiation processes. Phys. Rev..

[55] El-Oranya FAA, Wahiddinb MRB, Obadad A-SF (2008). Single-atom entropy squeezing for two two-level atoms interacting with a single-mode radiation field. Opt. Commun..

[56] Kuzmich A, Molmer K, Polzik ES (1997). Spin squeezing in an ensemble of atoms illuminated with squeezed light. Phys. Rev. Lett..

[57] Sanchez-Ruiz J (1995). Improved bounds in the entropic uncertainty and certainty relations for complementary observables. Phys. Lett. A.

[58] Iqbal S, Rivière P, Saif F (2010). Space-time dynamics of Gazeau-Klauder coherent states in power-law potentials. Int. J. Theor. Phys..

[59] Hall RL (1989). Spectral geometry of power-law potentials in quantum mechanics. Phys. Rev. A.

[60] Berrada K (2014). Improving quantum phase estimation via power-law potential systems. Laser Phys..

[61] Jena SN, Panda P, Tripathy TC (2000). Ground states and excitation spectra of baryons in a non-Coulombic power-law potential model. Phys. Rev. D.

[62] Jena SN, Rath DP (1986). Magnetic moments of light, charmed, and b-flavored baryons in a relativistic logarithmic potential. Phys. Rev. D.

[63] Berrada K, El Baz M, Hassouni Y (2011). Generalized Heisenberg algebra coherent states for power-law potentials. Phys. Lett. A.

[64] Agarwal GS (1988). Nonclassical statistics of fields in pair coherent states. J. Opt. Soc. Am. B.

[65] Zyczkowski K, Horodecki P, Sanpera A, Lewensteinm M (1998). Volume of the set of separable states. Phys. Rev. A.

[66] Vidal G, Werner RF (2002). Computable measure of entanglement. Phys. Rev. A.

[67] Riccardi A, Macchiavello C, Maccone L (2017). Tight entropic uncertainty relations for systems with dimension three to five. Phys. Rev. A.

[68] Abdalla MS, Obada A-SF, Abdel-Khalek S (2008). Entropy squeezing of time dependent single-mode Jaynes-Cummings model in presence of non-linear effect. Chaos Solitons Fract..

[69] Khalil EM, Abdalla MS, Obada A-SF (2006). Entropy and variance squeezing of two coupled modes interacting with a two-level atom: Frequency converter type. Ann. Phys..

[70] Fang M-F, Zhou P, Swain S (2000). Entropy squeezing for a two-level atom. J. Mod. Opt..

